# Complete genome sequence of a highly divergent astrovirus isolated from a child with acute diarrhea

**DOI:** 10.1186/1743-422X-5-117

**Published:** 2008-10-14

**Authors:** Stacy R Finkbeiner, Carl D Kirkwood, David Wang

**Affiliations:** 1 Departments of Molecular Microbiology and Pathology & Immunology, Washington University School of Medicine, St. Louis, MO, USA; 2 Enteric Virus Research Group, Murdoch Childrens Research Institute, Royal Children's Hospital, Victoria, Australia

## Abstract

**Background:**

Astroviruses infect a variety of mammals and birds and are causative agents of diarrhea in humans and other animal hosts. We have previously described the identification of several sequence fragments with limited sequence identity to known astroviruses in a stool specimen obtained from a child with acute diarrhea, suggesting that a novel virus was present.

**Results:**

In this study, the complete genome of this novel virus isolate was sequenced and analyzed. The overall genome organization of this virus paralleled that of known astroviruses, with 3 open reading frames identified. Phylogenetic analysis of the ORFs indicated that this virus is highly divergent from all previously described animal and human astroviruses. Molecular features that are highly conserved in human serotypes 1–8, such as a 3'NTR stem-loop structure and conserved nucleotide motifs present in the 5'NTR and ORF1b/2 junction, were either absent or only partially conserved in this novel virus.

**Conclusion:**

Based on the analyses described herein, we propose that this newly discovered virus represents a novel species in the family Astroviridae. It has tentatively been named Astrovirus MLB1.

## Background

Astroviruses are non-enveloped, single stranded, positive sense RNA viruses. Their genomes range from approximately 6 to 8 kb in length, are polyadenylated, and have both 5' and 3' non-translated regions (NTR) [1]. Their genomes have three open reading frames (ORFs) organized from 5' to 3' as follows: ORF 1a, which encodes a serine protease; ORF1b, which encodes the RNA dependent polymerase; and ORF 2, which encodes the structural proteins. A frameshift must occur during the translation of ORF1a in order for ORF1b to be translated. ORF 2 is translated from a sub-genomic RNA and produces a polyprotein which is cleaved by cellular proteases [1].

The family *Astroviridae *includes 8 closely related human serotypes as well as additional members that infect cattle, sheep, cats, dogs, deer, chickens, turkeys, and ducks [2]. Although some of the animal astroviruses are known to cause hepatitis or nephritis [3], astroviruses typically cause diarrhea in their hosts. Human astrovirus infections most frequently cause watery diarrhea lasting 2–4 days, and less commonly vomiting, headache, fever, abdominal pains, and anorexia in children under the age of 2, the elderly, and immunocompromised individuals [3]. The known human astroviruses account for up to ~10% of sporadic cases of non-bacterial diarrhea in children [4-8].

Diarrhea is the third leading infectious cause of death worldwide and is responsible for approximately 2 million deaths each year as well as [9] an estimated 1.4 billion non-fatal episodes [10,11]. In children, rotaviruses, caliciviruses, adenoviruses and astroviruses are responsible for the greatest proportion of cases [5,6,12-14]. Most epidemiological studies fail to identify an etiologic agent in ~40% of diarrhea cases [15-19]. Recently, we conducted viral metagenomic analysis of diarrhea samples using a mass sequencing approach with the explicit goal of identifying novel viruses that may be candidate causes of diarrhea. One of the stool samples we analyzed was collected in 1999 at the Royal Children's Hospital in Melbourne, Australia from a 3-yr old boy with acute diarrhea. Seven sequence reads were identified in this sample that shared ≤ 67% amino acid identity to known astrovirus proteins, suggesting that a novel astrovirus was present in the sample [20]. In this paper, we report the full sequencing and characterization of the genome of this astrovirus, referred to hereafter as astrovirus MLB1 (AstV-MLB1).

## Results and discussion

### Genome sequencing and analysis

In the previous metagenomic study [20], we identified seven sequence reads with limited identity to known astroviruses that could be assembled into two small contigs in a clinical stool sample. The contigs had 42–44%, and 59–61% amino acid identity to human astrovirus serine proteases and RNA-polymerases, respectively. In this study, the complete genome of the astrovirus present in the original stool specimen was sequenced to an average of >3× coverage [GenBank: FJ222451]. The virus has been tentatively named Astrovirus MLB1 (AstV-MLB1). Analysis of the genome showed that AstV-MLB1 has the same genomic organization as other astroviruses. Like other astroviruses, the AstV-MLB1 genome was predicted to encode three open reading frames (ORF1a, ORF1b, and ORF2) and contained both 5' and 3' non-translated regions (NTR), as well as a poly-A tail. The complete genome length of AstV-MLB1 was 6,171 bp, excluding the poly-A tail, slightly shorter when compared to other astrovirus genomes which range in size between ~6,400 and 7,300 bp [1]. A comparison of AstV-MLB1 genomic elements with those of fully sequenced astroviruses is shown in Table 1.

**Table 1:** Genome Comparison of AstV-MLB1 to other astroviruses

**Virus**	**Genome (bp)**	**5' UTR (bp)**	**ORF1a**	**ORF1b**	**ORF2**	**3' UTR**
Chicken AstV-1	6,927	15	3,017	1,533	2,052	305
Turkey AstV-1	7,003	11	3,300	1,539	2,016	130
Turkey AstV-2	7,325	21	3,378	1,584	2,175	196
Mink AstV	6,610	26	2,648	1,620	2,328	108
Ovine AstV	6,440	45	2,580	1,572	2,289	59
Human AstV-1	6,813	85	2,763	1,560	2,361	80
Human AstV-2	6,828	82	2,763	1,560	2,392	82
Human AstV-4	6,723	84	2,763	1,548	2,316	81
Human AstV-5	6,762	83	2,763	1,548	2,352	86
Human AstV-8	6,759	80	2,766	1,557	2,349	85
**AstV-MLB1**	**6,171**	**14**	**2,364**	**1,536**	**2,271**	**58**

The ORF 1a of astroviruses encodes a non-structural polyprotein which contains a serine-like protease motif. Pfam analysis revealed a region of ORF1a that has homology to a peptidase domain. In addition, alignment of AstV-MLB1 with other astroviruses revealed that AstV-MLB1 contains the amino acids of the catalytic triad (His, Asp, Ser) which are conserved in the 3C-like protease motif found in other viruses (data not shown) [21]. The residues RTQ which have been suggested to be involved in substrate binding are conserved among the human astroviruses, but vary in other viruses which have the 3C-like motif [21]. In AstV-MLB1, the predicted substrate binding residues (ATR) are identical to those found in *Ovine astrovirus *and not those of the human astroviruses (data not shown).

A second feature of astrovirus ORF1a is the presence of a bipartite nuclear localization signal (NLS) found in human, chicken, and ovine astroviruses, but not turkey astroviruses [22]. A bipartite NLS is characterized as having two regions of basic amino acids separated by a 10 aa spacer. The protein alignment of ORF1a revealed that AstV-MLB1 has a sequence motif similar to the putative NLS of human astroviruses. This region of the genome has also been predicted to potentially encode for a viral genome-linked protein (VPg) [23]. The high sequence similarity observed between AstV-MLB1 and other astroviruses in the motifs identified as essential for a putative VPg suggests that AstV-MLB1 may also encode a VPg (data not shown). While no experimental data exists supporting the prediction of the presence of a Vpg being encoded in any of the astrovirus genomes, we should note that we did encounter difficulty in obtaining the 5' end of the MLB1 genome until treatment of the RNA with proteinase K prior to RNA extraction was added to the experimental protocol.

Finally, the 2,364 nt sequence of AstV-MLB1 ORF1a is shorter than ORF1a sequences of other astroviruses, which range between ~2,500–3,300 nt (Table 1). The shorter length of AstV-MLB1 ORF1a relative to the human astroviruses is largely attributable to two deletions totaling 57 amino acids located within a highly conserved motif near the carboxyl terminus of human astroviruses 1–8. This deletion falls within a 144 aa region that has been mapped as being an immunoreactive epitope in human astroviruses [24] and is located in the non-structural protein p38 [21]. Recently, p38 has been reported to lead to apoptosis of the host cell which results in efficient virus replication [25] and particle release [26]. However, it is unclear how the genome deletion identified in AstV-MLB1 might influence these activities.

Astrovirus ORF1b is classically generated by a -1 ribosomal frameshift induced by the presence of a heptameric 'slippery sequence' (AAAAAAAC). [2]. A conserved slippery sequence was identified near the end of ORF1a of Ast-MLB1 and FSFinder was used to determine if the downstream sequence was capable of forming a stem-loop structure, as found in other astoviruses [27]. The predicted start position of ORF1b was then determined by selecting the first amino acid in frame with the slippery sequence. The 1b open reading frame of astroviruses encodes an RNA-dependent RNA polymerase (RNAP). Pfam analysis revealed that AstV-MLB1 ORF1b contains the RNA-dependent RNA polymerase domain found in other positive strand RNA viruses, suggesting this ORF does in fact encode for an RNAP.

Astrovirus ORF2 encodes a large structural polyprotein that is cleaved by cellular proteases to generate the viral capsid proteins. Following the convention of human astroviruses [28,29] by choosing a start codon for ORF2 located two nucleotides upstream of the ORF 1b stop codon resulted in a predicted protein length of 756aa. Pfam analysis of the predicted protein encoded by ORF2 identifies an astrovirus capsid motif, thereby congruent with the paradigm of astrovirus genome organization in which ORF2 encodes the structural capsid proteins.

The AstV-MLB1 ORF2 protein sequence was divided into four subregions for more detailed analysis as described [30]. Pair-wise comparisons of each region were conducted between the AstV-MLB1 sequence and the sequences of all astroviruses for which sequences were available. Consistent with previous reports, region I appeared to be the most conserved of the four regions and in each of the regions, AstV-MLB1 shared the most similarity to known human astroviruses. However, even in region I, AstV-MLB1 only exhibited 33–35% identity to known human astroviruses. In the less conserved regions II-IV, AstV-MLB1 shared only 5–27% amino acid identity to the known human astroviruses. By contrast, the range of identities between human astrovirus serotypes 1–8 were, 43–75%, 16–66% and 28–77% for regions II, III and IV, respectively. Overall, ASTV-MLB1 maintained higher conservation in region I of ORF2 than in other regions, consistent with paradigms established by analysis of other astroviruses.

### Non-coding features

**Figure 1:**
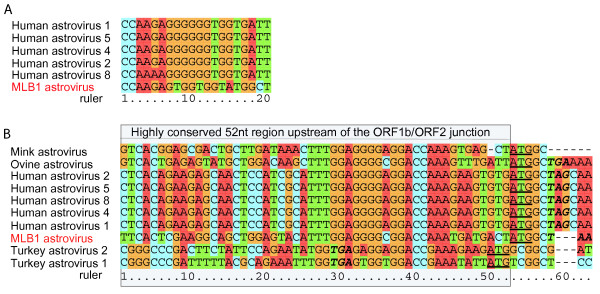
**Multiple sequence alignments of putative astrovirus regulatory regions**. A.) Alignment of the 20 nucleotides at the very 5' end of the Astrovirus MLB1 genome with those of fully sequenced astroviruses. MLB1 only shares 13 of the 20 conserved nucleotides present in human strains 1–8. B.) Alignment of the 52 nt highly conserved nucleotide motif (shown in box) present immediately upstream of the ORF1b/ORF2 junction of Astrovirus MLB1 and other astroviruses. (Note: there is no overlap in the Turkey Astroviruses). MLB1 lacks the high degree of sequence identity seen between the human astroviruses. The start codon of ORF2 is shown underlined and the stop codon of ORF1b is shown italicized in bold for each virus.

Multiple independent 5' RACE experiments were performed to determine the precise 5' end of the genome. Based on these experiments, the AstV-MLB1 5' NTR was determined to be 14 nt long. This is similar in length to the ~10–20 nt 5'NTRs of avian astroviruses [1], but much shorter than the 80–85 nt long 5'NTRs of the 8 human astrovirus serotypes (Table 1). Notably, the human astroviruses share a 20 nt consensus sequence at the terminal 5' nucleotides of the genome which is not conserved in other astroviruses (data not shown). AstV-MLB1 contained 13 out of the 20 consensus nucleotides, including the most 5'CCAA motif within the this region [31] (Fig. 1A). These data support the notion that the sequence we generated does contain the very 5' terminus of the genome.

Human astroviruses contain a 120 nt region at the junction between ORF1b and ORF2 that is ~95–97% conserved between serotypes [32]. The most highly conserved core 52 nt region of this sequence is 99–100% identical among the human astrovirus serotypes. The exact role of this sequence is not known, but it is hypothesized to be a regulatory element of the sub-genomic RNA that encodes for ORF2. Alignment between AstV-MLB1 and other human astroviruses of the highly conserved 52 nt at the ORF1b/ORF2 junction revealed that AstV-MLB1 possessed only 61.5% identity in this region (Fig. 1B). By contrast, the known animal astroviruses share only 44–59.6% identity in this 52 nt region with human astroviruses as determined by pair-wise comparisons. Interestingly, AstV-MLB1 shares 71.2% identity in this region to *Ovine Astrovirus*.

All of the previously described astroviruses, with the exception of turkey astrovirus 2, have a conserved RNA secondary structure referred to as the stem-loop II-like motif (s2m) found at the 3' end of the genome in the 3' NTR [33]. This motif is also present in some coronaviruses and equine rhinovirus serotype 2. Mutations within this motif are generally accompanied by compensatory mutations that restore base pairing [33]. The conservation of such a sequence motif across multiple viral families suggests that it may play a broad role in the biology of positive stranded RNA viruses [33]. The exact function of this stem loop is not known, but it is hypothesized to interact with viral and cellular proteins needed for RNA replication. Nucleotide alignment of the 150 nucleotides at the 3' terminus of the AstV-MLB1 genome and other viruses known to contain the stem-loop motif suggested that AstV-MLB1 does not have this conserved nucleotide motif (data not shown). Furthermore, it also has the shortest 3'NTR reported to date for an astrovirus. (Table 1) [1].

### Phylogenetic analysis

**Figure 2:**
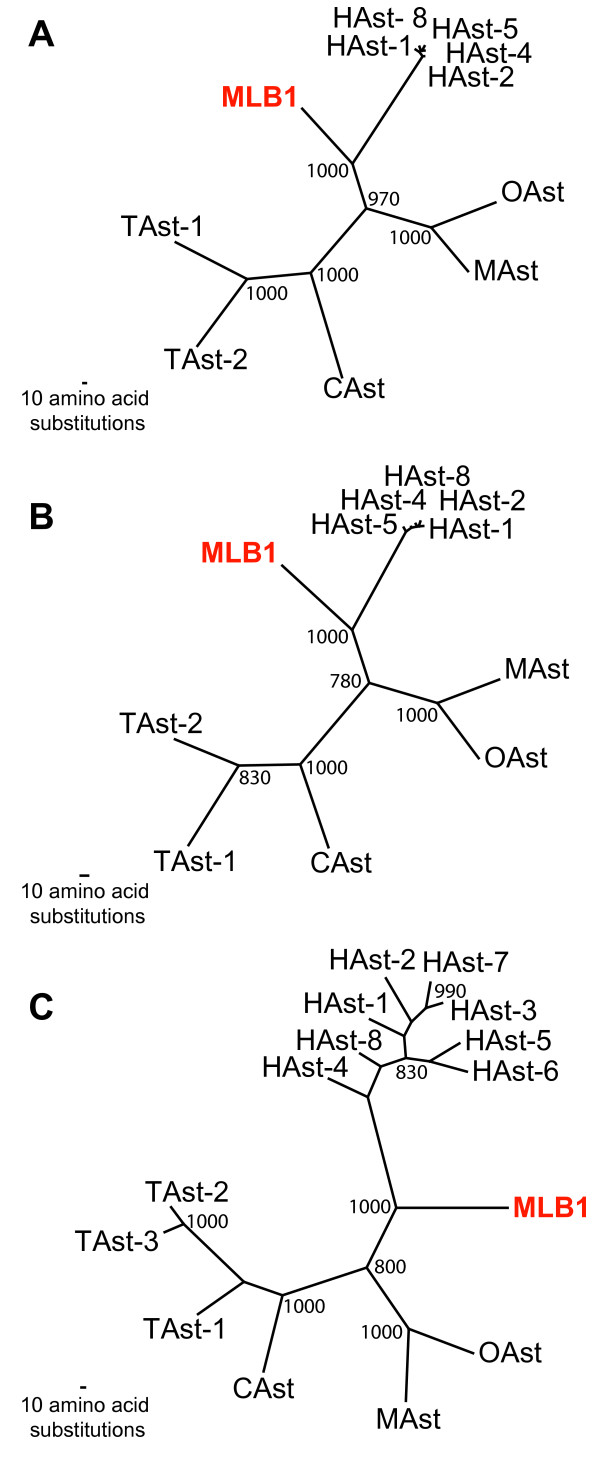
**Phylogenetic analysis of AstV-MLB1 open reading frames**. Phylogenetic trees are based on amino acid sequences and were generated using the maximum parsimony method with 1,000 bootstrap replicates. Significant bootstrap values are shown. (A) ORF1a; (B) ORF1b; (C) ORF2. HAstV = Human astrovirus; CAstV = Chicken astrovirus; MAstV = Mink astrovirus; TAstV = Turkey astrovirus; OAstV = Ovine astrovirus.

Multiple sequence alignments of the three astrovirus open reading frames were performed and bootstrapped maximum parsimony trees were generated (Fig. 2). The trees confirmed initial assessments that AstV-MLB1 is a novel astrovirus[20]. The trees for ORFs 1a and 1b (Fig. 2a, b) both indicated that AstV-MLB1 is most closely related to the human astroviruses, although it is highly divergent from them. AstV-MLB1 ORF1a only has 9–28% amino acid identity to other astrovirus ORF1a proteins and the pairwise sequence alignments of ORF1b revealed 35–54% amino acid identity between ORF1b proteins of AstV-MLB1 and other astroviruses (Table 2). The maximum parsimony tree for ORF2 (Fig. 2c) shows that there is greater divergence among all of the sequences for ORF2, as is to be expected of the capsid region. However it is still evident that AstV-MLB1 is quite divergent from any of the known human astroviruses. Based on the predicted 756aa protein of ORF2, AstV-MLB1 has only 11–24% amino acid identity to other astrovirus capsid precursor proteins (Table 2).

**Table 2:** Comparison of astrovirus proteins to predicted AstV-MLB1 proteins

**ORF**	**Est. Size (aa)**	**% Amino Acid Identity to:**													
		HAstV-1	HAstV-2	HAstV-3	HAstV-4	HAstV-5	HAstV-6	HAstV-7	HAstV-8	TAstV-1	TAstV-2	TAstV-3	ChAstV-1	OAstV	MAstV
1a	787	28	28	NA	29	29	NA	NA	29	9	9	NA	10	22	24
1b	511	54	54	NA	54	54	NA	NA	54	36	35	NA	36	47	44
2	756	24	24	24	23	23	24	24	24	15	16	16	11	18	19

### Origin of virus

At this point, the origin of AstV-MLB1 is unclear. AstV-MLB1 may be a bona fide human virus capable of infecting and replicating within the human gastrointestinal tract that had evaded detection until now. Alternately, it may be a passenger virus present simply as a result of dietary ingestion, as has been described previously for plant viruses detected in human stool [34]. Of course, viruses derived from dietary intake that appear to cause human disease, such as Aichi virus, have been described previously [35,36]. Another possibility is that this virus may represent zoonotic transmission from some other animal species that is the true host for Astrovirus MLB1. Traditionally it has been thought that astroviruses have a strict species tropism. However, recent evidence has emerged that suggests that interspecies transmission does occur. For example, chicken astrovirus antibodies have been detected in turkeys [37] and an astrovirus was isolated from humans whose capsid sequence most closely resembled that of feline astrovirus[1]. Because of the uncertainty as to the identity of the true host species and the host range for this virus, we have tentatively named this novel virus Astrovirus MLB1 (AstV-MLB1). Efforts to define whether AstV-MLB1 is a novel human pathogen are underway.

## Conclusion

Complete sequencing and genome analysis of Astrovirus MLB1 revealed that the virus has three open reading frames sharing the same organization as other astroviruses. Phylogenetic analysis of the open reading frames clearly demonstrated that AstV-MLB1 is highly divergent from any of the known astroviruses. Furthermore, AstV-MLB1 lacks the conservation seen between human astroviruses 1–8 in the non-translated regions of the genome such as the 5' and 3' NTR and the ORF1b/2 junction. The aggregate analysis of the non-coding features and ORFs as well as the phylogentic analysis clearly indicates that AstV-MLB1 is highly divergent from all previously described astroviruses.

The divergence of AstV-MLB1 from known astroviruses in the non-translated regions of the genome is particularly interesting because these regions are nucleotide motifs that are thought to play regulatory roles in viral replication. This suggests that AstV-MLB1 may behave very differently from the known astroviruses and that additional studies on the regulation of AstV-MLB1 transcription and replication may broaden our understanding of astrovirus paradigms.

Astroviruses are associated with diarrhea predominantly in young children and immunocompromised individuals. The discovery of AstV-MLB1 in a liver transplant patient fits well with the known clinical parameters of astrovirus infection. We previously reported that the only other virus detected in this stool was a TT virus [20], which is thought to be non-pathogenic [38]. It is therefore tempting to speculate that AstV-MLB1 is the pathogenic agent that caused this case of diarrhea. However, whether AstV-MLB1 is a bona fide human virus capable of causing diarrhea will have to be established by further experimentation and epidemiological surveys.

## Methods

### Specimen

A stool sample was collected from a 3 year old boy admitted to the Royal Children's Hospital with acute diarrhea in 1999. The child had previously undergone a liver transplant one year prior to this episode of diarrhea, however the immunological status was unknown.

### RNA extraction

RNA was isolated from the primary stool filtrate using RNA-Bee (Tel-Test, Inc.) according to manufacturer's instructions. In some cases, the stool filtrate was treated with 2.5 mg\ml proteinase K (Sigma) for 30 min prior to RNA extraction.

### Genome amplification and sequencing

The astrovirus sequence reads previously detected in the primary stool filtrate [20] [GenBank accessions: ET065575, ET065576, ET065577, ET065579, ET065580, ET065581, ET065582] were assembled into two contigs, and the nucleic acid between the contigs was obtained by RT-PCR. For reverse transcription reactions, cDNA was generated with MonsterScript RT at 65°C and amplified with Taq (Invitrogen). Subsequent 5' and 3' RACE reactions were done to obtain the entire genome. To generate high quality sequence coverage, 7 pairs of specific primers that spanned the complete genome in overlapping ~1 kb fragments were used in RT-PCR reactions and then cloned and sequenced using standard Sanger sequencing chemistry. All amplicons were cloned into pCR4.0 (Invitrogen). These 7 primer pairs were used to confirm the sequence of the viral genome from both the primary stool sample and the passage 2 tissue culture sample. The complete genome sequence of AstV-MLB1 has been deposited in [GenBank: FJ222451].

### ORF prediction and annotation

Open reading frames 1a and 2 were predicted for AstV-MLB1 using the NCBI ORF Finder program. ORF1b was predicted based on the frameshift paradigm that occurs in other astroviruses by identifying a heptameric slippery sequence [39]. Conserved motifs were identified using Pfam [40].

### Pair-wise alignments

Bioedit was used to determine the percent identity between sequences as determined by pair-wise alignments.

### Phylogenetic analysis

ClustalX (1.83) was used to carry out multiple sequence alignments of the protein sequences associated with all three of the open reading frames of representative astrovirus types. Maximum parsimony trees were generated using PAUP with 1,000 bootstrap replicates [41]. Available nucleotide or protein sequences of the following astroviruses were obtained: Human Astrovirus 1 [GenBank: NC_001943]; Human Astrovirus 2 [GenBank: L13745]; Human Astrovirus 3 [GenBank: AAD17224]; Human Astrovirus 4 [GenBank: DQ070852]; Human Astrovirus 5 [GenBank: DQ028633]; Human Astrovirus 6 [EMBL: CAA86616]; Human Astrovirus 7 [Gen Bank: AAK31913]; Human Astrovirus 8 [GenBank: AF260508]; Turkey Astrovirus 1 [GenBank: Y15936]; Turkey Astrovirus 2 [GenBank: NC_005790]; Turkey Astrovirus 3 [GenBank: AY769616]; Chicken Astrovirus [GenBank: NC_003790]; Ovine Astrovirus [GenBank: NC_002469]; and Mink Astrovirus [GenBank: NC_004579].

## Competing interests

The authors declare that they have no competing interests.

## Authors' contributions

DW conceived and designed the experiments. SF carried out the experiments and analysis. CK contributed reagents/materials. SF and DW wrote the paper.
